# Supplementary material for: End Sequence Analysis Toolkit (ESAT) expands the extractable information from single-cell RNA-seq data
Source: Genome Res. 2016 Oct;26(10):1397–410. doi: 10.1101/gr.207902.116 (PMC5052061; doi:10.1101/gr.207902.116)
Supplement: Supplemental Material [file supp_gr.207902.116_Supp_Materials.tar.gz › ESAT-esat_v0.1/doc/html/dir_7f9c8a639ce9225e124ad15182dc5540.html]

ESAT: java/umms/core/annotation Directory Reference


ESAT


- Main Page
- Related Pages
- Packages
- Classes
- Files

All Classes Namespaces Files Functions Variables Enumerator Pages

- java
- umms
- core
- annotation

annotation Directory Reference

Directory dependency graph for annotation:

|  |  |
| --- | --- |
| Directories | |
| directory | filter |
|  | |

|  |  |
| --- | --- |
| Files | |
| file | AbstractAnnotation.java |
|  | |
| file | AbstractAnnotationCollection.java |
|  | |
| file | Annotation.java |
|  | |
| file | AnnotationCollection.java |
|  | |
| file | AnnotationFileReader.java |
|  | |
| file | AnnotationList.java |
|  | |
| file | BasicAnnotation.java |
|  | |
| file | CompoundInterval.java |
|  | |
| file | Gene.java |
|  | |
| file | PSLRecord.java |
|  | |
| file | SingleInterval.java |
|  | |


---

Generated on Fri Aug 15 2014 15:11:15 for ESAT by  

 1.8.7
